# Microstructure Transitions and Dry-Wet Spinnability of Silk Fibroin Protein from Waste Silk Quilt

**DOI:** 10.3390/polym11101622

**Published:** 2019-10-08

**Authors:** Xin Zhang, Zhijuan Pan

**Affiliations:** 1 College of Textile and Clothing Engineering, Soochow University, Suzhou 215021, China; zx18860901672@163.com; 2 National Engineering Laboratory for Modern Silk, Soochow University, Suzhou 215123, China

**Keywords:** waste silk, regenerated silk fibroin, conformation transition, quantitative analysis, dry-wet spinning

## Abstract

With excellent biocompatibility and biodegradability, silk fibroin has been developed into many protein materials. For producing regenerated silk fibroin (RSF) fibers, the conformation transition of silk fibroin needs to be thoroughly studied during the spinning process. Since the many silk fabrics that are discarded comprise an increasing waste of resources and increase the pressure on the environment, in this paper, waste silk fiber was recycled in an attempt to prepare regenerated fibroin fiber by dry-wet spinning. Ethanol was the coagulation bath. The rheological properties of all the RSF solutions were investigated to acquire rheology curves and non-Newtonian indexes for spinnability analysis. Four stages of the spinning process were carried out to obtain RSF samples and study their conformation transitions, crystallization, and thermal properties by Fourier transform infrared spectroscopy (FTIR), X-ray diffraction, and differential scanning calorimetry. Quantitative analysis of the FTIR results was performed to obtain specific data regarding the contents of the secondary structures. The results showed that higher concentration spinning solutions had better spinnability. As the spinning process progressed, random coils were gradually converted into β-sheets and crystallization increased. Among the different influencing factors, the ethanol coagulation bath played a leading role in the conformation transitions of silk fibroin.

## 1. Introduction

Bombyx mori silk is known as the "queen of fibers" because of its outstanding mechanical properties compared with most nature fibers. Silk fiber is a polymer composed of fibroin and glue-like sericin. Sericin, with the function of cohesion, coats the twin fibroin filaments together. After extraction from degummed silk fibers, firoin protein can be developed into various morphologies, including gels, sponges, films [1], and fibers, and combined with diverse functional materials to produce extraordinary properties. Currently, fibroin has broad application prospects in biomedical materials, such as tissue engineering scaffold materials [2,3] and fixed active material sensors [4], due to the excellent biocompatibility and tunable biodegradability [5] of this protein by virtue of its unique compositions and structure [6,7,8,9].

However, silk resources are very limited and precious due to their long production cycle and high cost. To solve this problem, many scholars have tried to extract silk fibroin to fabricate regenerated fibroin fibers. By simulating the spinning method of silkworms, people have expected to prepare superior regenerated silk fibroin fibers with an adjustable morphology and specific structure; further exploring the forming process and mechanisms of natural silk fibers could also promote significant development in the field of silk fibroin materials. Nevertheless, the performance of regenerated silk fibroin fibers developed based on current technical methods is still no match for that of natural silk fibroin fibers [10,11,12]. The relevant research on bionic protein fibers shows the importance of studying the relationship between protein conformation and function and the mechanism of the conformation transformation. 

The secondary structure of silk fibroin has been extensively discussed relative to its structural transformation [13,14,15]. Common prominent secondary structures include random coils, α-helices, β-sheets, and β-turns [16]. Random coils are characteristic conformations of macromolecules and are dominant in silk fibroin aqueous solution. The peptide chain of the α-helix coils into a spiral around an imaginary central axis that is maintained by the hydrogen bonds within the chain. These two structures are related to the elasticity of silk fibroin fibers. In the β-sheet structure, multiple peptide chains or several peptides in a chain are arranged in parallel by oxygen bonds, and the serrated main chain forms a folded sheet, which is related to the strength of the silk fibroin fiber. As frequently occurring secondary structures in polypeptides, β-turns with common 180° loops are composed of four amino acids [17].

Shimizu first reported the presence of two types of crystals in fibroin [18]. Later, Kratki et al. named these crystals Silk I and Silk II [19]. Currently, the crystalline structure of silk protein is mainly divided into an amorphous structure (Silk I) dominated by random coils and α-helices and a stable structure (Silk II) dominated by β-sheets [20,21,22].

The secondary structures mentioned above are frequently detected when Fourier transform infrared spectroscopy (FTIR) [23,24,25] is used to characterize the construction transformation. FTIR, the earliest method used for this purpose, is quite practical and simple for detection of the peptide bonds of protein molecules containing various amide bonds. The amide I–III bands, including the secondary structure of protein molecules, can be very sensitive to detect. The amide I region is often used in quantitative analysis of the secondary structure with the second derivative, deconvolution, and curve fitting [26,27,28,29,30]. The structural changes in fibroin can be analyzed according to the crystallinity obtained by X-ray diffraction [31,32] and differential scanning calorimetry.

During the process of producing silk fabrics, there is a certain amount of waste material. In addition, large amounts of household silk textiles are discarded in garbage cans every year. In recent years, as the concept of the circular economy has grown, the public have begun to pay attention to the growing amounts of waste textiles. Germany, Britain, France, the United States, and Japan have passed legislative rules for the recycling of waste textiles and have established recycling systems. Since silk fibroin has the extraordinary characteristics mentioned above and is expensive due to a complex production process and high market demands, besides, using silk fabrics wastes may decrease breeding of silkworms, many scholars have carried out research related to the recycling and reuse of silk. To more effectively recycle and reuse waste silk, certain scientists treated waste silk by impregnation and melting without damaging the internal molecular composition to prepare composites [33,34,35,36]. Certain researchers found methods to destroy the molecular structure of waste silk by high temperature and high pressure or chemical reagent treatment, and successfully obtained silk sericin or fibroin protein [37,38].

Therefore, we tried to fabricate regenerated silk fibroin (RSF) fibers with fibroin extracted from waste silk fabric using dry-wet spinning equipment constructed in-house. Ethanol was chosen for the coagulation bath, and spinning solutions were prepared at four different concentrations. Since the spinnability of the fibroin from the RSF aqueous solutions still needed to be improved, the spinning solutions were tested by rheological measurement to obtain the non-Newtonian index (n). The apparent morphology, secondary structure, crystallization, and thermal properties of the RSF samples acquired at different stages were tested by scanning electron microscopy (SEM), Fourier transform infrared spectroscopy (FTIR), X-ray diffraction (XRD), and differential scanning calorimetry (DSC).

## 2. Materials and Methods

### 2.1. Materials

Waste silk was obtained from discarded silk quilts used for over eight years. Ethanol was purchased from Chinasun Specialty Products Co., Ltd. (Jiangsu, China). The other chemical reagents, such as LiBr and Na_2_CO_3_, were analytical grade and used without further purification.

### 2.2. Preparation of RSF Aqueous Solutions 

The waste silk was degummed by boiling in 0.2 wt% Na_2_CO_3_ aqueous solution for 25 min and then was washed several times with deionized water. The waste silk fibroin was allowed to air dry overnight at room temperature. A 10% (w/v) solution of degummed waste silk fibroin in 9.3 mol/L LiBr aqueous solution was prepared by heating to 60 °C for 1 h with continuous stirring. After dialysis against deionized water by a cellulose semipermeable membrane (MWCO: 14 kDa) at room temperature for 3 days to remove salts, the RSF aqueous solution was centrifuged at 6000 r/min for 6 min to remove impurities. RSF aqueous solutions with the desired concentrations, i.e., 19, 22, 25 and 28 wt%, were prepared by dialysis against a 15% (w/v) polyethylene glycol (PEG) solution.

### 2.3. Dry-Wet Spinning of RSF Aqueous Solutions

The silk fibroin fibers were spun through dry-wet spinning equipment constructed in-house. The solutions were extruded through a spinneret 0.26 mm in diameter at room temperature using a syringe and pump. Through the air gap, the solution went into the 100% ethanol coagulation bath. The extrusion flow rate was 5 mL/min. 

### 2.4. Preparation of Test Samples

According to the spinning process, four stages of silk fibroin proteins were selected for exploring the conformation transformation: (1) the spinning solutions, (2) the spinning solutions extruded through the spinneret before contacting the coagulation bath, (3) the spinning solutions just entering the coagulation bath, and (4) the spinning solutions treated by the coagulation bath for a certain amount of time. Stages (1) and (2) were liquid RSF samples with different concentrations that were cast on polyethylene plates at 25 °C to form films. Stages (3) and (4) were deformed coagulated samples or RSF fibers formed from the various concentrations that were dried overnight at 25 °C after washing by deionized water to remove the residue from the coagulation bath. The 16 sample codes are shown in Table 1.

## 3. Characterization and Evaluation

The Fourier transform infrared spectroscopy (FTIR) spectra were obtained on a Nicolet iS5 spectrophotometer (Thermal Fisher Scientific, Madison, WI, USA) in the wavenumber range of 800–1800 cm^−1^. A total of 16 scans were accumulated with a resolution of 4 cm^−1^ for each spectrum. All the FTIR spectra presented in this work are smoothed absorption data, corrected by background calibration and normalization.

The rheology of all the solutions was measured on a rotational rheometer (Discovery HR-2, TA Instruments, New Castle, DE, USA). The 40 mm diameter cone plate geometry was used for the solutions. The chosen gap was 26 μm for all the solutions. Continuous ramp step experiments were examined at a shear-rate range of 0.1 to 100 s^−1^ at 25 °C. Each sample was made and tested three times to avoid errors.

The crystalline structure of all the samples were performed by X-ray diffraction (XRD) using the X’ Pert Pro MPD X-ray diffraction system (PAN analytical, Eindhoven, Holland) with a CuKα radiation source (wavelength, λ = 0.154 nm) of 40 kV and 30 mA. The XRD diffraction spectra were recorded in the 2θ range 5–45° at a scan rate of 3°/min.

**Figure 1:**
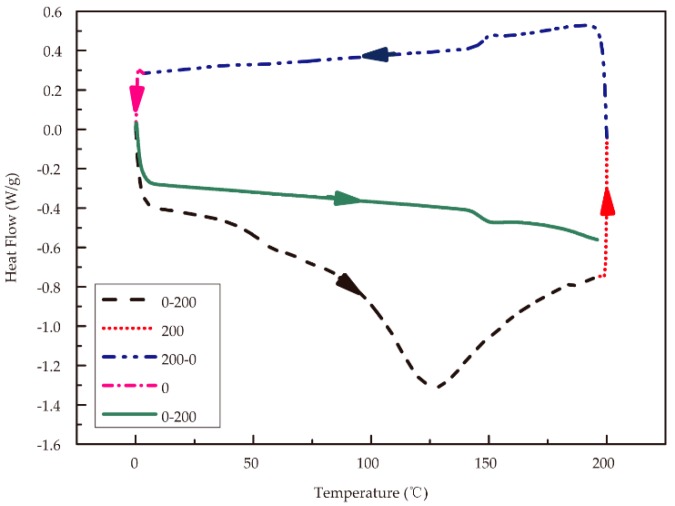
Heat-cool-reheat differential scanning calorimetry (DSC) scan process for RSF samples.

Differential scanning calorimetry (DSC) was performed on approximately 5 mg of material in an open crucible using a Q2000 calorimeter (TA Instruments, New Castle, DE, USA) under nitrogen flow. A heating rate of 20 °C/min was used for standard DSC analysis. The DSC process is shown in Figure 1. The temperature was raised from 0 to 200 °C (black dashed line), held for 2 min at 200 °C (red dotted line), and decreased to 0 °C (blue dashed and dotted line) at the same rate to eliminate the influences of moisture and internal stress in the samples. After the isothermal temperature was maintained for 2 min (magenta dashed and dotted line), the temperature was increased to 200 °C again (green solid line) at the rate of 20 °C/min to obtain an accurate glass transition temperature.

Images of the RSF fibers were taken on a scanning electron microscope (SEM) (S4800, Hitachi, Tokyo, Japan) at a voltage of 3 kV.

## 4. Results and Discussion

### 4.1. Rheological Behaviors of RSF Solutions with Various Concentrations

**Figure 2:**
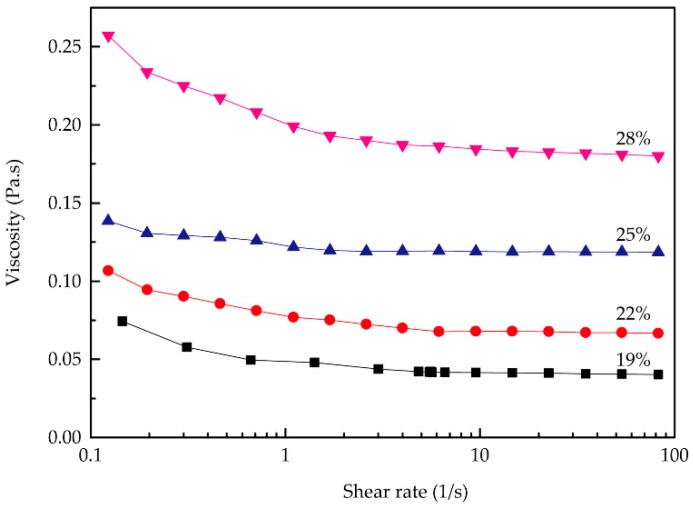
Rheological curves of the RSF solutions with different concentrations.

In Figure 2, the viscosity of the RSF aqueous solutions decreased with increasing shear rate, and the solutions exhibited typical shear thinning behavior with variation of the shear rate, indicating that all the solutions behaved as non-Newtonian fluids.

All the spinning solutions could be characterized by a power-law equation [39]:

Τ = K^·^γn (1)

where τ represents shear stress, ^·^γ is the shear rate, K is the consistency coefficient, and n is the non-Newtonian index. The logarithm of both sides of Equation (1) was taken, yielding Equation (2) as follows:

Lgτ = K + nlg^·^γ (2)

**Figure 3:**
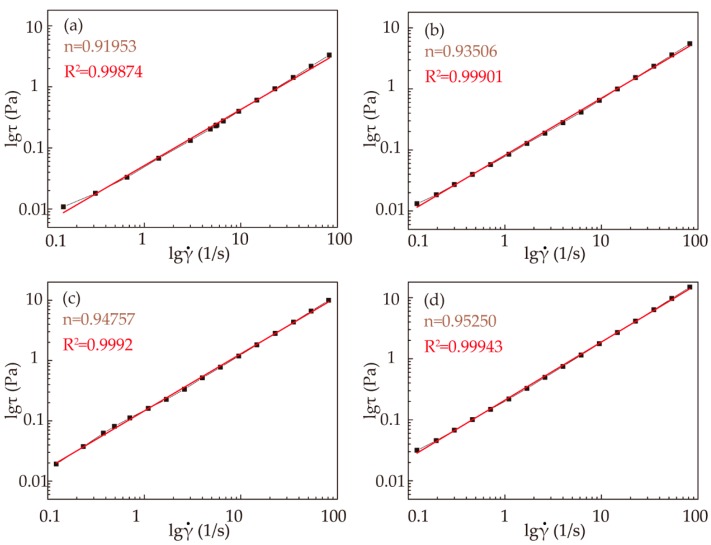
Rheological behavior fitting results of RSF solutions with different concentrations: (**a**) 19%; (**b**) 22%; (**c**) 25%; (**d**) 28%.

Comparisons of the estimated (red lines) and experimental (line and squares) lgτ−lg^·^γ curves are shown in Figure 3 for various concentrations of RSF solutions:

The fitting results of n values for the RSF solutions are shown in Figure 3. All the fluids studied had n < 1, indicating that all the fibroin solutions were pseudoplastic fluids. With increasing solution concentration, n approached 1, indicating that the fluidity and spinnability of the RSF solutions increased. However, it takes a longer time to obtain a higher concentration of the solution, and as the concentration increases, the RSF solution is prone to deformation. Therefore, obtaining the spinning solution with a concentration over 28% is more difficult. 

### 4.2. Conformation Analysis of RSF Samples from FTIR Spectra

FTIR spectroscopy was used to study the conformational states of the RSF samples. The FTIR absorptions of silk fibroin conformations are reported in Table 2 [40,41].

**Figure 4:**
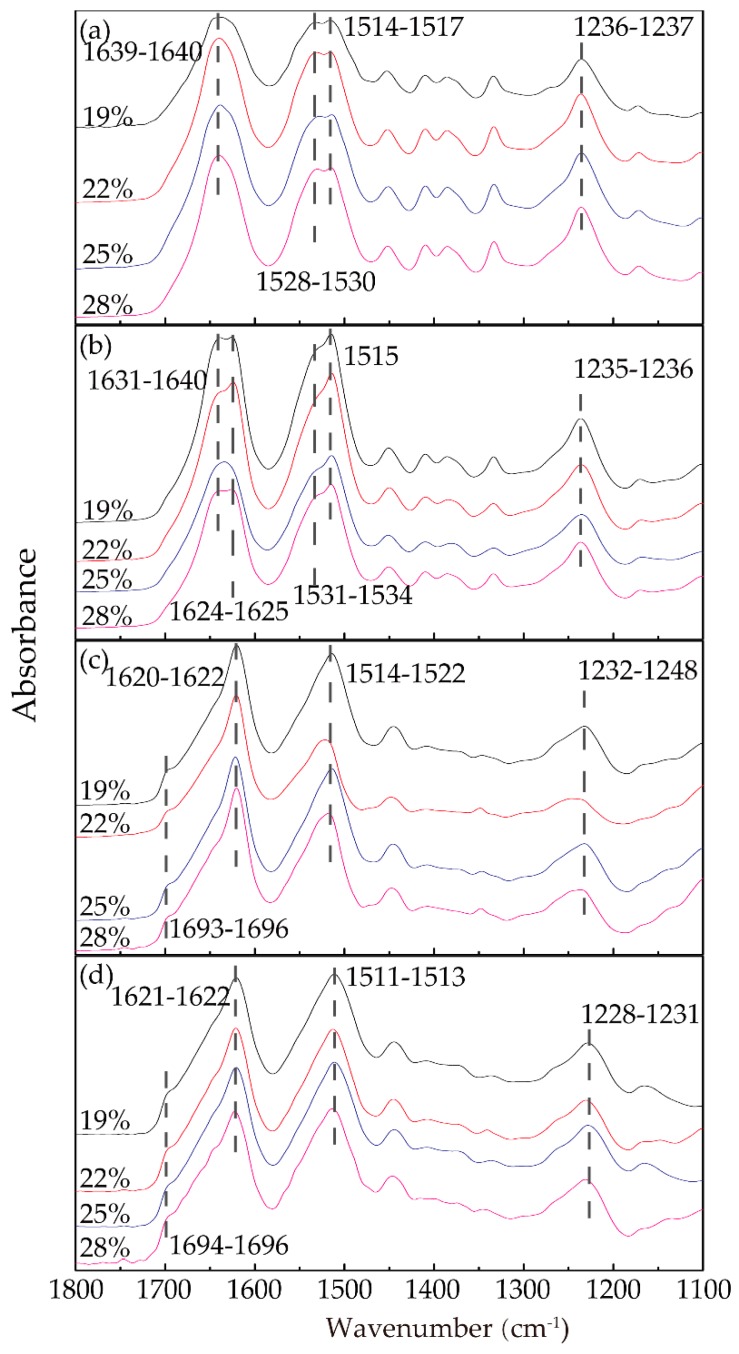
FTIR spectra of all the samples from the amide I to amide III bands: (**a**) F1; (**b**) F2; (**c**) E1; (**d**) E2.

Figure 4 shows the infrared absorption spectra of the RSF samples with different concentrations in four stages, displaying amide I, II, and III absorption bands. As shown in Figure 4a, the trends of the four spectra were mostly similar; the RSF films exhibited absorption bands at 1514–1517 cm^−1^ (amide II), corresponding to the β-sheet conformation, and 1639–1640 cm^−1^ (amide I), 1528–1530 cm^−1^ (amide II), and 1236–1237 cm^−1^ (amide III), corresponding to the random coil conformation. According to Arrondo et al. [42], the spectral bands between 1620 and 1625 cm^−1^ were the interchain structures, while the spectral bands between 1630 and 1637 cm^−1^ were the intrachain structures. Comparing Figure 4a,b, after extrusion through the spinneret into the air gap, the amide I peaks of samples in Figure 4b obviously shifted to lower wavenumbers (from 1639–1640 cm^−1^ to 1624–1625 cm^−1^), remaining shoulders at 1631–1640 cm^−1^. The intensity of the shoulder at approximately 1531–1534 cm^−1^ of amide II decreased slightly compared with the similar peaks in Figure 4a, while the peaks in amide III scarcely shifted from 1236–1237 cm^−1^ to 1235–1236 cm^−1^, corresponding to the random coil conformation.

As shown in Figure 4c, after entering the ethanol coagulation bath, the characteristic peaks of amides I, II, and III all underwent distinct wave shifts to lower wavenumbers, while the shoulder peaks of amides I and II disappeared and the width of characteristic peaks narrowed. It was noteworthy that, in Figure 4c,d, the obvious shoulders at 1693–1695cm^−1^ and 1694–1695cm^−1^ respectively appeared corresponding to antiparallel structure [43], showing that ethanol greatly influenced the conformation of the RSF. The amide II peaks, compared with Figure 4b, shifted from 1531–1534 cm^−1^ to 1514–1522 cm^−1^, corresponding to random coils and β-sheets [44,45,46], respectively. This result confirmed that the ethanol had a strong ability to change the silk fibroin from the random coil conformation to the β-sheet conformation. The position and number of peaks in Figure 4d were slightly different than those of Figure 4c, while the peaks of amides II and III shifted slightly to lower wavenumbers, meaning that the coagulation bath treatment time had a certain effect on the structural transformation. 

### 4.3. Estimation of β-sheet Content in Samples Using Amide I Band from FTIR Spectra

**Figure 5:**
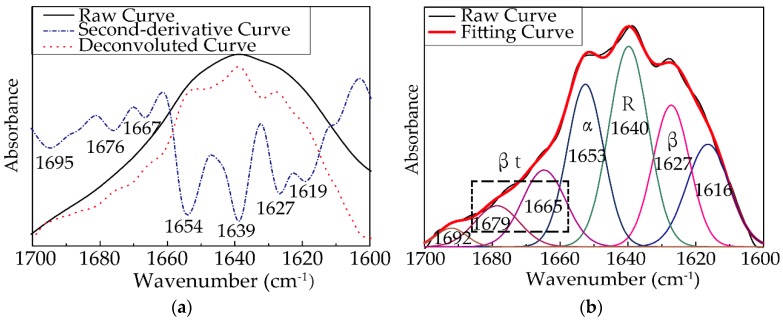
(**a**) Raw curve (black line), deconvoluted curve (red dotted line), and second-derivative curve (blue dashed and dotted line) of the amide I absorption band of 25-F1 sample; (**b**) The quantitative analysis of the amide I band for 25-F1 sample (the black line is the original spectrum, the red line is the fitting spectrum, and the colorful lines are the contributions to the spectra from each type of secondary structure).

The amide I band (1600–1700 cm^−1^) was selected for quantitative analysis of the secondary structure of RSF samples [42]. The spectra were smoothed to remove the influence of vapor. The second-derivative spectra were calculated to obtain the positions of the secondary structures, and the deconvolution spectra were acquired by the conservative values of a full width at half maximum (FWHH) of 13.0 cm^−1^ and a resolution enhancement factor of 2.4 to reverse the effects of convolution on the recorded data, as shown in Figure 5. Peak fitting (Origin) software (v4.12) was used to fit the curve of the deconvolution spectrum of the sample with the Gauss and Lorentz Amp functions until the fitting degree was close to 1, as shown in Figure 5. After adding up the area of sub-spectra corresponding to each secondary structure and calculating the percentage of the area occupied by these sub-spectra, respectively, the content (%) of each secondary structure can be calculated quantitatively.

**Figure 6:**
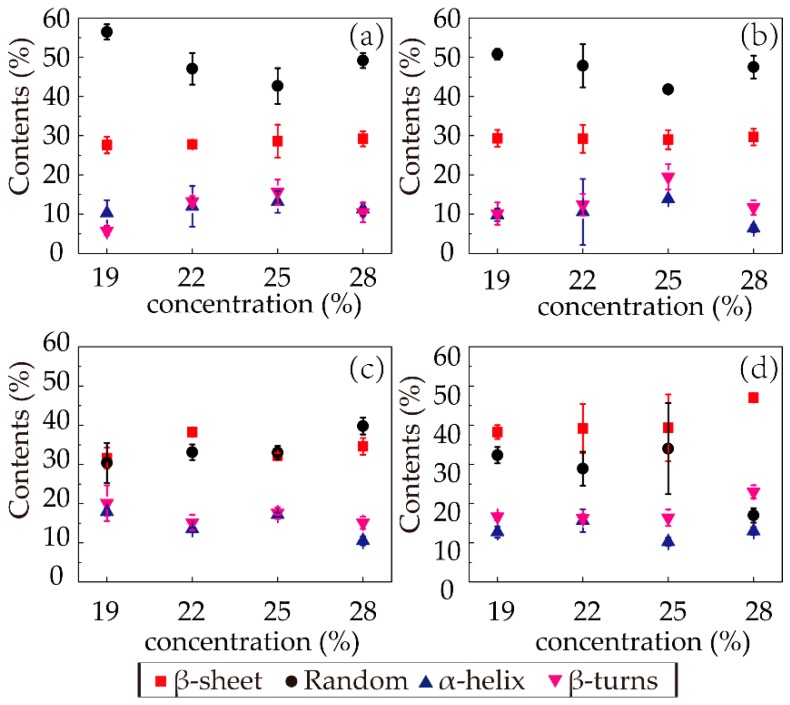
Contents of secondary structures in the RSF samples: (**a**) F1; (**b**) F2; (**c**) E1; (**d**) E2.

The relative intensity of the spectra of amide I provided the fractional secondary structure contents of random coils (black circles), β-sheets (red squares), α-helices (blue triangles), and β-turns (magenta inverted triangles) in each sample, shown in Figure 6. In the first (F1) and second (F2) stages, the secondary structure distributions were similar. The dominant secondary structure was random coils, followed by β-sheets. With increasing concentration, the random coil content first decreased and then increased as the concentration increased to 28%, and the β-sheet content did not vary much with concentration in the first two stages, while the α-helix and β-turn contents were similarly low.

In the third stage (E1), the random coil contents of the samples with different concentrations all significantly decreased (from 50.76, 47.83, 41.80, and 47.49% to 30.38, 33.09, 33.01, and 39.77%, respectively), with obvious increases in the β-sheet contents (from 29.31, 29.21, 28.99, and 29.64% to 31.59, 38.22, 32.23, and 34.61%). This phenomenon confirmed that the coagulation had a strong ability to transform the structure from random coil to β-sheet. Regarding the fourth stage (E2), the trends of decreasing random coil content and increasing β-sheet content with increasing concentration were distinct, while the α-helix and β-turn contents experienced slight irregular fluctuations. Since the β-sheet content was relevant to the performance, the β-sheet distribution in the last stage was noteworthy. With increasing concentration, the β-sheet content gradually increased with an increment from 34.61 to 47.00% in sample 28-E2. This result indicated that the spinning solutions with high concentrations better facilitated the formation of β-sheets of silk fibroin protein through ethanol coagulation in the same amount of time.

### 4.4. Crystallinity Analysis of Silk Fibroin Protein Samples

#### 4.4.1. X-Ray Diffraction (XRD) 

**Figure 7:**
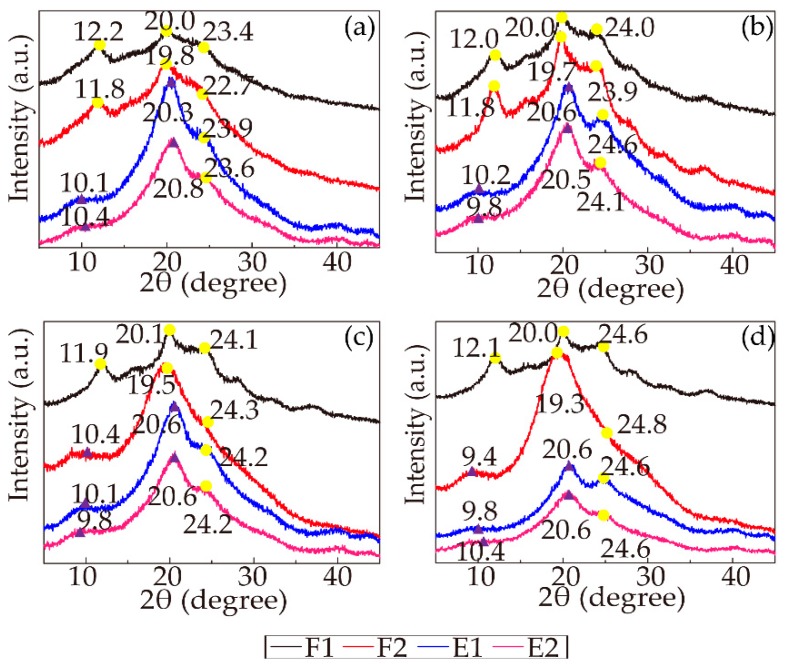
X-ray spectra of all the RSF samples (the yellow points represent Silk I, and the purple triangles represent Silk II): (**a**) 19%; (**b**) 22%; (**c**) 25%; (**d**) 28%.

As the most well-known secondary structures, Silk I and Silk II have been extensively analyzed by XRD. Silk I, corresponding to the α-helix conformation, is the metastable structure of fibroin. Silk II, corresponding to the β-sheet structure, is a conformation where all the molecular chains run in alternate directions and form stable antiparallel chain pleated sheets. The transformation from Silk I to Silk II has always been regarded as an important indicator of β-sheet crystallization. Silk I shows three main diffraction peaks at approximately 2θ = 12.2, 19.7, and 24.7°, while Silk II diffraction peaks are located at approximately 2θ = 9.1° and 20.7° [47]. The XRD spectra of the RSF samples are presented in Figure 7. Taking samples 19-F1, 19-F2, 19-E1, and 19-E2 as examples, sample 19-F1 exhibited three main diffraction peaks at 2θ = 12.2, 20.0, and 23.4°, assigned to Silk I, and these three peaks of shifted slightly to 11.8, 19.8 and 22.7°, respectively, for sample 19-F2 but were still the characteristic peaks of Silk I. Nevertheless, Silk II diffraction peaks started to emerge in samples 19-E1 (10.1 and 20.3°) and 19-E2 (10.4 and 20.8°), with only one Silk I diffraction peak remaining at 23.9° and 23.6° in these two stages, respectively. This phenomenon suggested that the crystallinity of the samples treated by ethanol increased. Additionally, for samples with different concentrations, the trends of the peak shifts were incredibly similar; in the first stage, all three characteristic peaks represented Silk I, and in the second stage, most of the peaks shifted but remained Silk I. As the concentration increased, the characteristic peaks of samples 25-F2 and 28-F2 started to shift to Silk II diffraction peaks, meaning that high concentration improved the crystallinity. In the last two stages, all the samples with various concentrations showed distinct Silk II diffraction peaks, while the position of the Silk I diffraction peaks at approximately 24.7° did not change much among all the stages. The number of miscellaneous peaks decreased, and the curves smoothed as the spinning progressed. The aforementioned results showed that all the stages increased the crystallinity and increasing concentration positively impacted the crystallinity. The ethanol strongly induced the Silk II (β-sheet) structure, improving the crystallinity.

#### 4.4.2. Differential Scanning Calorimetry (DSC)

**Figure 8:**
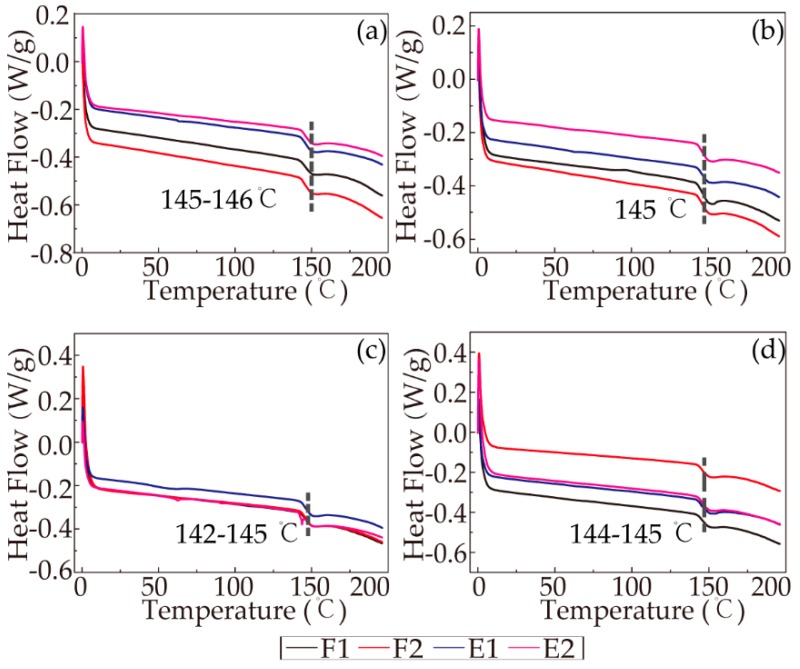
DSC curves of the cool-reheat scans: (**a**) 19%; (**b**) 22%; (**c**) 25%; (**d**) 28%.

The heat-cool-reheat process was used to evaporate the intermolecular bound water and obtain an accurate glass transition temperature. A clear glass transition temperature could be detected during the last run of the DSC curve (cool-reheat), and the glass transition temperatures of all the RSF samples are reported in Figure 8. The curves clearly show that the differences in the glass transition temperatures (T_g_) of all the sample were negligible, meaning that the thermodynamic stabilities of the samples were similar.

**Figure 9:**
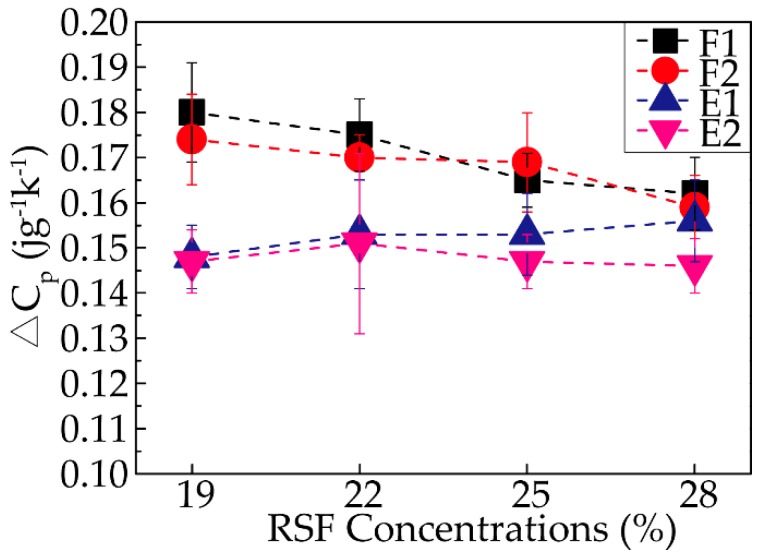
Variations in the heat capacities (△c_p_) of all the RSF samples.

In addition to the glass transition temperature, the variation in the heat capacity (△c_p_) at T_g_ was a key measurement. Because △c_p_ is proportional to the amorphous content in the sample, an increase in the crystallinity results in a decrease in the △c_p_. Thus, the degree of crystallinity in the samples could be roughly determined based on the following equation:

(3) $$△c_{p}=|\frac{{Ф}_{2}-{Ф}_{1}}{m\beta }|$$

where Ф1 and Ф2 represent the onset and end of the glass transformation from the DSC signal (heat flows), respectively, m is the sample quality, and β is the heating rate. The distinctive specific results of all the samples are exhibited in Figure 9; with increasing concentration, △c_p_ decreased for almost every stage except the third stage (E1), as demonstrated by sample 28-E1, which showed with the increasing concentration, the degree of crystallization became higher to some extent. With the progression of the spinning process, the △c_p_ of all the samples decreased except for sample 25-F2. In addition, the degree of the decrease in the △c_p_ was most significant between F2 and E1, followed by ethanol coagulation treatment and air gap treatment. In summary, the ethanol coagulation played an important role in promoting the crystallinity of the silk fibroin, while the four stages all affected the crystallinity to an extent. The conclusions were consistent with the results mentioned above.

### 4.5. Morphology of the Coagulated Samples

**Figure 10:**
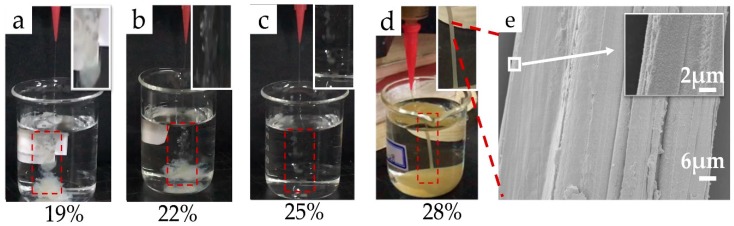
(**a**–**d**) Effect of concentration on the dry-wet spinnability of the RSF solutions; (**e**) Morphology of the RSF fibers (28-E2).

Figure 10 shows images of the spinning conditions of the RSF solutions treated by the ethanol coagulation bath (Figure 10a–d). The effect of concentration during dry-wet spinning of the RSF fibers was obvious in the ethanol coagulation bath. Figure 10a shows that the solution quickly diffused into the coagulation bath; Figure 10 b shows that the solution coagulated discontinuously; Figure 10c shows that bead shaped RSF started to form. The low concentration solution had difficulties forming fibers in the ethanol coagulation bath, while the 28% RSF solution successfully formed continuous fibers, as shown in Figure 10d. The experimental results were consistent with the rheological test results showing that increasing concentration increased the spinnability. SEM images of the surface of sample 28-E2 are shown in Figure 10e at 1500× and 10,000×. The fibers displayed rough morphology with obvious and irregular grooves, possibly from the drafting process. The surface of the as-spun fibers was rough. Further improvement is still needed to optimize the apparent morphology of the fibers.

## 5. Conclusions

In this paper, silk fibroin was extracted from waste silk to prepare a regenerated fibroin solution. To study the spinnability of this kind of RSF solution, dry-wet spinning was used to attempt to fabricate regenerated silk fibroin fibers with a device constructed in-house. Adjusting the concentrations of the RSF solutions, spinning solutions with concentrations of 19, 22, 25, and 28 wt% were obtained to study the effect of the concentration on spinnability. The coagulation bath was a 100% ethanol solution. According to the steps of the spinning process, RSF samples from the spinning solution, air gap, and ethanol coagulation bath for a short period of time, and from the ethanol coagulation bath for a long period of time were chosen to investigate the conformation transitions of silk fibroin. Rheological measurements showed that all the spinning solutions behaved as non-Newtonian fluids and that an increasing concentration improved spinnability according to the non-Newtonian index. Comparing the FTIR results of all the RSF samples, coagulation in ethanol was able to induce silk fibroin to transform from the random coil to the β-sheet conformation, followed by the concentration of the spinning solution, the air gap, and coagulation treatment time. The XRD results and DSC proved the conclusions. Due to the low viscosity of the RSF solutions extracted from the waste silk, the spinning conditions were not good. Only when the concentration reached 28 wt% was the spinning solution able to present continuous fibers in the ethanol coagulation bath. However, the surface of the RSF fibers was rough and damaged, and much work remains to optimize the spinnability of the RSF solution.

## Figures and Tables

**Table 1:** Sample codes of various concentrations of regenerated silk fibroin (RSF) from 4 stages of the spinning process.

Sample Code ^1^	Concentration/wt%	Stage	Sample Code ^1^	Concentration/wt%	Stage
19-F1	19	(1)	25-F1	25	(1)
19-F2	19	(2)	25-F2	25	(2)
19-E1	19	(3)	25-E1	25	(3)
19-E2	19	(4)	25-E2	25	(4)
22-F1	22	(1)	28-F1	28	(1)
22-F2	22	(2)	28-F2	28	(2)
22-E1	22	(3)	28-E1	28	(3)
22-E2	22	(4)	28-E2	28	(4)

^1^ The number in the code represents the concentration of the spinning solution; F1: the sample was in film form in the first stage; F2: the sample was in film form in the second stage; E1: the sample was in ethanol coagulation in the third stage; E2: the sample was in ethanol coagulation in the fourth stage.

**Table 2:** Infrared absorptions of silk fibroin conformations.

Fibroin Conformation	Amide I /cm^−1^	Amide II /cm^−1^	Amide III /cm^−1^
β-sheet	1622–1637	1515–1525	1265
α-helix	1656–1662	1545	1240
Random coil	1638–1655	1535–1545	1235
